# Gut-Associated Plasmacytoid Dendritic Cells Display an Immature Phenotype and Upregulated Granzyme B in Subjects with HIV/AIDS

**DOI:** 10.3389/fimmu.2015.00485

**Published:** 2015-09-24

**Authors:** Sergei V. Boichuk, Svetlana F. Khaiboullina, Bulat R. Ramazanov, Gulshat R. Khasanova, Karina A. Ivanovskaya, Evgeny Z. Nizamutdinov, Marat R. Sharafutdinov, Ekaterina V. Martynova, Kenny L. DeMeirleir, Jan Hulstaert, Vladimir A. Anokhin, Albert A. Rizvanov, Vincent C. Lombardi

**Affiliations:** ^1^Kazan State Medical University, Kazan, Russia; ^2^Institute of Fundamental Medicine and Biology, Kazan Federal University, Kazan, Russia; ^3^Nevada Center for Biomedical Research, Reno, NV, USA; ^4^Republican Infectious Disease Hospital, Kazan, Russia; ^5^Kazan City Clinical Hospital #7, Kazan, Russia; ^6^Department of Gastroenterology, Kazan City Hospital #12, Kazan, Russia; ^7^Himmunitas Institute, Brussels, Belgium; ^8^Department of Gastroenterology, General Hospital Jan Portaels, Vilvoorde, Belgium; ^9^Department of Biochemistry and Molecular Biology, School of Medicine, University of Nevada, Reno, NV, USA

**Keywords:** HIV/AIDS, plasmacytoid dendritic cell, pDC, gut, granzyme B

## Abstract

Plasmacytoid dendritic cells (pDCs) in the periphery of subjects with human immunodeficiency virus (HIV)/acquired immunodeficiency syndrome (AIDS) decrease over time, and the fate of these cells has been the subject of ongoing investigation. Previous studies using animal models as well as studies with humans suggest that these cells may redistribute to the gut. Other studies using animal models propose that the periphery pDCs are depleted and gut is repopulated with naive pDCs from the bone marrow. In the present study, we utilized immunohistochemistry to survey duodenum biopsies of subjects with HIV/AIDS and controls. We observed that subjects with HIV/AIDS had increased infiltration of Ki-67^+^/CD303^+^ pDCs, a phenotype consistent with bone marrow-derived pre-pDCs. In contrast, Ki-67^+^/CD303^+^ pDCs were not observed in control biopsies. We additionally observed that gut-associated pDCs in HIV/AIDS cases upregulate the proapoptotic enzyme granzyme B; however, no granzyme B was observed in the pDCs of control biopsies. Our data are consistent with reports in animal models that suggest periphery pDCs are depleted by exhaustion and that naive pDCs egress from the bone marrow and ultimately infiltrate the gut mucosa. Additionally, our observation of granzyme B upregulation in naive pDCs may identify a contributing factor to the gut pathology associated with HIV infection.

## Introduction

Human immunodeficiency virus (HIV) infection is characterized by a rapid loss of peripheral CD4^+^ T lymphocytes during the acute phase (1, 2). CD4^+^ T-cell levels partially recover after this phase; however, they gradually decline as the infection progresses, ultimately contributing to the development of acquired immunodeficiency syndrome (AIDS). In order to establish an infection, the viral GP120 envelope glycoprotein must bind to the cell’s primary CD4 receptor as well as a coreceptor, either CCR5 or CXCR4 (3, 4). In addition to T cells, macrophages and dendritic cells also express these receptors and are thus susceptible to HIV infection (5, 6). It is therefore not surprising that decreased numbers of dendritic cells are observed in the blood of HIV cases and their presence inversely correlate with plasma viral load. For example, Grassi and coworkers reported that conventional dendritic cells (cDCs) expressing the integrin CD11c showed a significant decline during the course of HIV infection and did not return to normal levels after highly active antiretroviral therapy (HAART) (7). Loss of cDCs as well as plasmacytoid dendritic cells (pDCs) during HIV infection was also reported by Donaghy et al. (8) and similar observations were described by Pacanowski coworkers (9). Consistent with these observations, Azzoni et al. reported that cDC and pDC levels were lower in HIV viremic children when compared with those with undetectable plasma viral load (10). In the same study, they showed that subjects with declining levels of CD4^+^ T cells are more likely to present with lower DC numbers when compared with those with a stable CD4^+^ T cell population. These data support a characteristic pathology of HIV infection where declining DC levels inversely correlate with plasma viral load and further suggest that DCs play an important role in HIV pathogenesis.

Plasmacytoid DCs are the primary source of type I IFN and account for over 95% of all IFNα produced by circulating lymphocytes (11). Through the inhibition of viral replication, IFNα would likely play a central role in controlling HIV replication and consistent with this supposition, previous studies have reported that high levels of serum IFNα are often found in asymptomatic HIV carriers (12). In contrast, severe cases of AIDS have been associated with a diminished capacity to produce IFNα and the development of opportunistic infections (13). Furthermore, decreased IFNα levels are reported to correlate with lower circulating pDC numbers (14). Collectively, these data suggest that a decline in circulating pDCs and/or a diminished functional capacity is associated with the lower serum IFNα levels observed in subjects with HIV/AIDS.

A decline in peripheral pDCs during the course of HIV infection is well documented; however, the mechanism responsible for this decline remains a matter of current investigation. *In vitro* studies have shown that pDCs are susceptible to HIV infection and readily support viral replication (15). For this reason, their decline has been suggested to be the result of cytopathic viral replication (16). It has also been proposed that this decline may be the result of redistribution into the tissue, such as the regional lymph nodes (17, 18). Indeed, pDC redistribution into the lymph nodes has been observed in the early stages of simian immune deficiency virus (SIV) infection, the animal model for human HIV infection (17, 19, 20). Nevertheless, depletion of pDCs in lymphoid nodes of subjects infected with HIV has been documented (21), suggesting that the potential site of pDC redistribution remains elsewhere. Recently, Lehmann and colleagues observed pDC accumulation into the gut mucosa of HIV-infected subjects (22). Consistent with observations in SIV-infected macaques, pDCs in HIV cases increased their expression of gut-homing receptors (6, 23), implying that depletion of circulating pDCs is likely a result of their redistribution. However, a recent report by Bruel et al. suggests that the loss of pDCs in the periphery is the result of pDC exhaustion and the apparent redistribution of pDCs to the gut can be explained as pDC precursors migrating from the bone marrow to the gut (24). In a follow-up study to an early report (23), Li et al. observed that, in acute SIV infection of rhesus macaques, gut-homing was imprinted upon pDCs in the bone marrow, which resulted in a decline in pDCs from circulation and secondary lymphoid tissue and subsequent accumulation of hyperfunctional CD4^+^ pDCs in the mucosae (25).

In the present study, we have investigated the distribution and phenotype of pDCs in human subjects with HIV/AIDS. Consistent with previous reports, our data show a statistically significant decline in circulating pDCs in cases when compared with healthy controls. Using immunohistochemistry, we also observed a significant increase of pDC infiltration into the duodenal mucosal tissue of HIV cases when compared with control biopsies. Additionally and consistent with observations made by Bruel et al. in SIV-infected cynomolgus macaques, we observed that duodenal-associated pDCs in HIV-positive subjects express the cellular proliferation marker Ki-67. Our study supports the previous report of Bruel et al. (24) and Li et al. (25); however, further studies will be required to fully appreciate the contribution of this subpopulation of pDCs to the enteropathy of HIV infection.

## Materials and Methods

### Subjects

Twenty-three subjects (15 males and 8 females) who were hospitalized at the Republican Center for AIDS Prophylaxis and Prevention, Republic of Tatarstan, were enrolled in this study. Diagnosis of HIV infection was established based on the presence of anti-HIV antibodies using ELISA and confirmed by Western blot. Blood samples were collected from the 23 HIV cases and six of these subjects consented to providing a duodenal biopsy for analysis. Additionally, blood samples were collected from 16 healthy donors. For control biopsies, we utilized duodenal biopsies from eight individuals who underwent routine gastroscopy for gastritis and were otherwise healthy. The Institutional Review Board of the Kazan Federal University approved this study and informed consent was obtained from each study subject according to the guidelines approved under this protocol (article 20, Federal Law “Protection of Health Right of Citizens of Russian Federation” N323-FZ, 11.21.2011) and in accord with the Declaration of Helsinki (2008). Surplus clinical biopsies from subjects with gastritis were acquired under an exemption to IRB as determined by the University of Nevada, Reno Office of Research Integrity (exemption #508962-1).

### Clinical presentation of HIV cases

Diagnosis of HIV infection was established by detection of anti-HIV antibodies by ELISA and confirmed by Western blot. A total of 23 HIV cases were enrolled in this study (Table 1); eight of whom were female (35%) and 15 (65%) were male with an average age of 35.6 years (range, 21–46 years). Mode of transmission included sexual contact (11 cases; 48%) and IV drug users (12 cases; 52%). Enrolled were 12 cases with the severe form (52%), 3 cases with the advanced form (13%), 4 cases with the mild form (17.5%), and 4 cases with non-significant form (17.5%). Sixteen cases (70%) received antiviral treatment while seven cases (30%) remained without virus-specific therapy. Antiviral treatment included nucleoside analogs, non-nucleoside reverse transcriptase inhibitors, and protease inhibitors. Average HIV RNA viral load was 4412.1 ± 342 copies/ml, where 16 cases (70%) had a viral load higher than 250 copies/ml; the remaining seven cases (30%) had an undetectable viral load or less than 250 copies/ml. The mean CD4^+^ T-cell count was 306.1 ± 56 cells/mm^3^. As the disease progressed, some subjects were diagnosed with opportunistic infections, among which were candidiasis, hairy leukoplakia, and tuberculosis. HIV case characteristics and status are summarized in Table 1 and Table S1 in Supplementary Material.

**Table 1:** **Demographics of HIV cases by World Health Organization (WHO) immunological classification**.

WHO HIV-associated immunological classification	CD4 (cells/mm^3^)	Sex		Total	
		
		Male	Female	*n*	%
Severe	<200	8	4	12	52.0
Advanced	200–349	3	0	3	13.0
Mild	350–499	1	3	4	17.5
None/not significant	≥500	3	1	4	17.5
Total		15 (65%)	8 (35%)	23	100

### Antibodies for flow cytometry and immunohistochemistry

PerCP mouse anti-human CD45 was from Beckman Coulter (Brea, CA, USA). PE Anti-Ki-67 (clone B56) and PE mouse IgG1,κ isotype control were from BD Pharmigen (San Jose, CA, USA); PE mouse anti-human GZMB IgG1 (clone GB11) and PE mouse IgG1 isotype control were from Life Technologies (Carlsbad, CA, USA). FITC or APC mouse anti-human CD303 (clone AC144); PE mouse anti-human CD123 (clone AC145); PE mouse anti-human CD80 (clone 2D10); PE mouse anti-human CD56 IgG1 (clone AF12-7H3); APC mouse anti-human CD11c IgG2b (clone MJ4-27G12); APC mouse IgG2b isotype control; and PE mouse anti-human CD4 (clone VIT4) were from MiltenyiBiotec (Auburn, CA, USA).

### Flow cytometry analysis

Anticoagulated blood was collected in EDTA-containing vacutainer tubes by venipuncture. Whole blood (100 μl) was labeled with anti-human CD45-PerCP, anti-human CD303-APC, and anti-human CD123-PE for 20 min at room temperature, lysed with FACS Lyse (BD Biosciences, San Jose, CA, USA), and analyzed immediately on a FACS Canto II flow cytometer using FACS Diva software (BD Biosciences). A minimum of 300,000 events was collected for each sample and pDCs were identified as being CD45^+^CD303^+^CD123^+^.

### Tissues preparation and immunohistochemical analysis

Limitations on gut tissue samples made flow cytometery analysis impractical; therefore, we characterized duodenum biopsies by immunohistochemistry. Fresh tissues were fixed in 4% paraformaldehyde for 4 h at 4°C and cryoprotected with a 30% sucrose solution in phosphate-buffered saline (PBS) before being paraffin embedded. Immunohistochemical (IHC) staining was performed on 2–3-μm-thick tissue sections. Tissue slides were deparaffinized with xylene and rehydrated through a graded alcohol series. Antigen retrieval was carried out by boiling slides in sodium citrate (0.01M, pH 6.0) at 95°C for 10 min. The slides were next rinsed in PBS and incubated in cold methanol for 20 min at −20°C. Tissue sections were then incubated with human AB serum to block non-specific staining (1 h at 37°C) and then incubated with the labeled antibody overnight at 4°C in a humidified chamber. Slides were then washed (3×; Tween 0.1% PBS) and examined using a Leitz TCS-SP2 RS scanning laser confocal microscope (Wetzlar, Germany) and images were captured with Leitz analysis software.

In order to identify duodenum-infiltrating pDCs, we utilized a panel of fluorescently labeled monoclonal antibodies specific for putative surface cell receptors know to be expressed on mature and naive pDCs. Included in this panel were monoclonal antibodies specific for CD123, which is normally expressed on all mature and BM-derived pre-pDCs; CD303, which is uniquely expressed on circulating pDCs (26) and also on BM-derived pre-pDCs as early as Stage II (27); Ki-67, to distinguish naive pDCs (Ki-67^+^) from mature non-dividing (Ki-67^−^) pDCs; and the costimulatory marker, CD80, which is not expressed on BM-derived pre-pDCs at any stage, was used as an activation marker. We additionally probed our biopsies for the myloid CD marker, CD11c, which is also expressed on a minor population of BM-derived pDCs, and finally, in order to identify pDCs with cytotoxic potential, we probed our specimens for granzyme B (GZMB), and CD56.

### Statistical analysis

Data are presented as mean/SD. Statistical analysis was performed using Mann–Whitney test for comparisons between individual experimental groups (case and control). Significance was established at a value of *p* < 0.05.

## Results

### Decreased pDCs in the periphery of HIV/AIDS case

**Figure 1:**
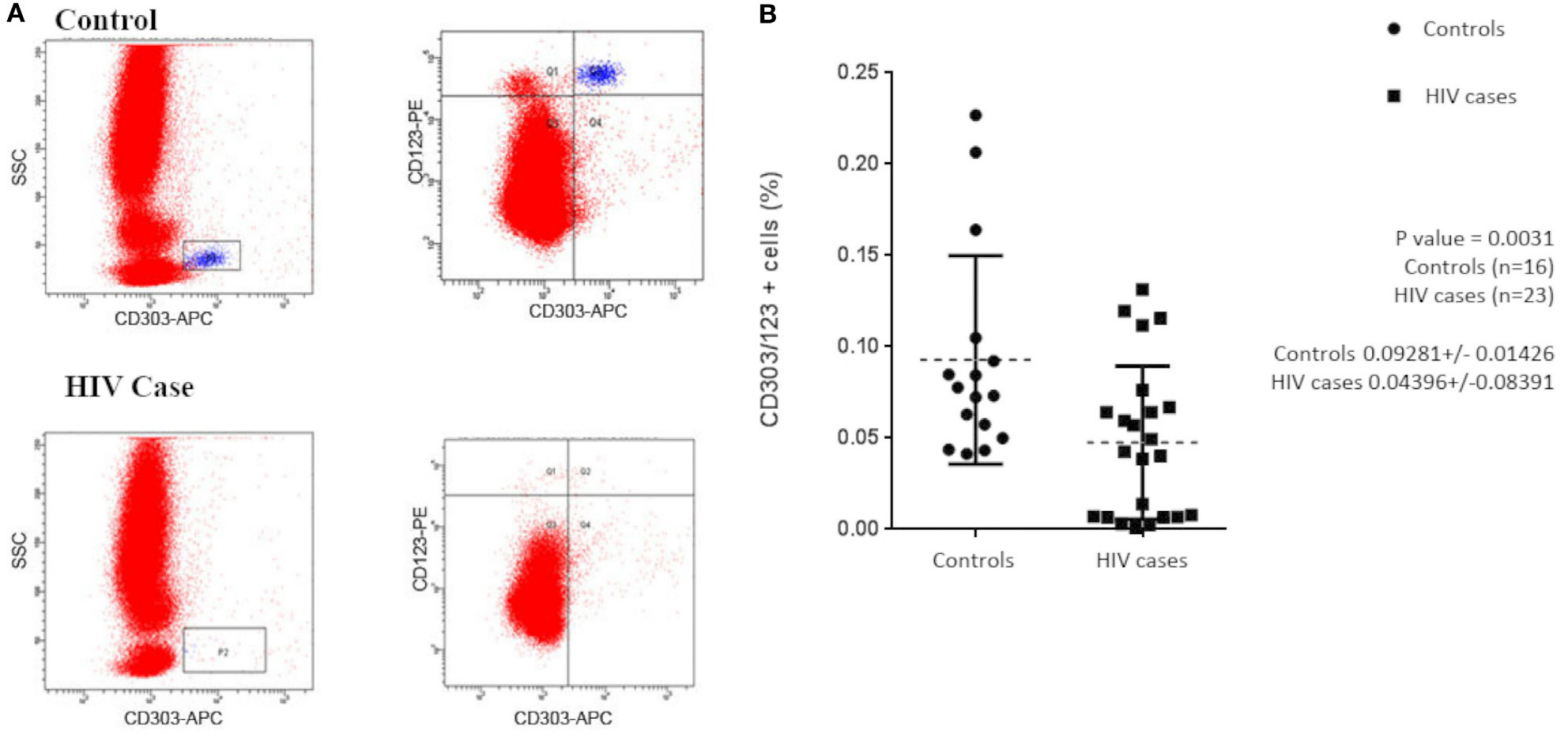
**(A)** Flow cytometry was used to evaluate circulating plasmacytoid dendritic cells (pDCs) in HIV/AIDS cases and controls. Aliquots of whole blood from 23 cases and 16 controls were labeled with APC-anti-CD303, PE-anti-CD123, and PerCp-anti-CD45. Lymphocytes were initially gated by CD45^+^/side scatter and pDCs were identified as CD303^+^/CD123^+^ lymphocytes. **(B)** pDCs, as a percentage of total lymphocytes, were used to evaluate differences between cases and controls. Mean pDC counts of HIV cases were 0.044 ± 0.084 and controls were 0.093 ± 0.014 (*p* = 0.0031).

Previous studies have shown that subjects with HIV/AIDS have decreased circulating pDCs when compared with healthy controls. To confirm that our study population was consistent with those previously reported, we initially evaluated our subjects for circulating pDCs by flow cytometry. Total lymphocytes were first gated by CD45^+^/side scatter (data not shown) and then pDCs were identified as a population of CD303^+^/CD123^+^ lymphocytes (Figure 1A). The mean pDC count was calculated as a ratio of CD303^+^CD123^+^CD45^+^ over total CD45^+^ lymphocytes. Consistent with the observations of others, we observe that, on average, subjects with HIV/AIDS had significantly lower circulating CD303^+^CD123^+^ lymphocytes when compared with healthy controls, 0.044 ± 0.084 vs. 0.093 ± 0.014 (*p* = 0.0031) (Figure 1B).

### Increased pDC infiltration of the duodenum in association with HIV infection

**Figure 2:**
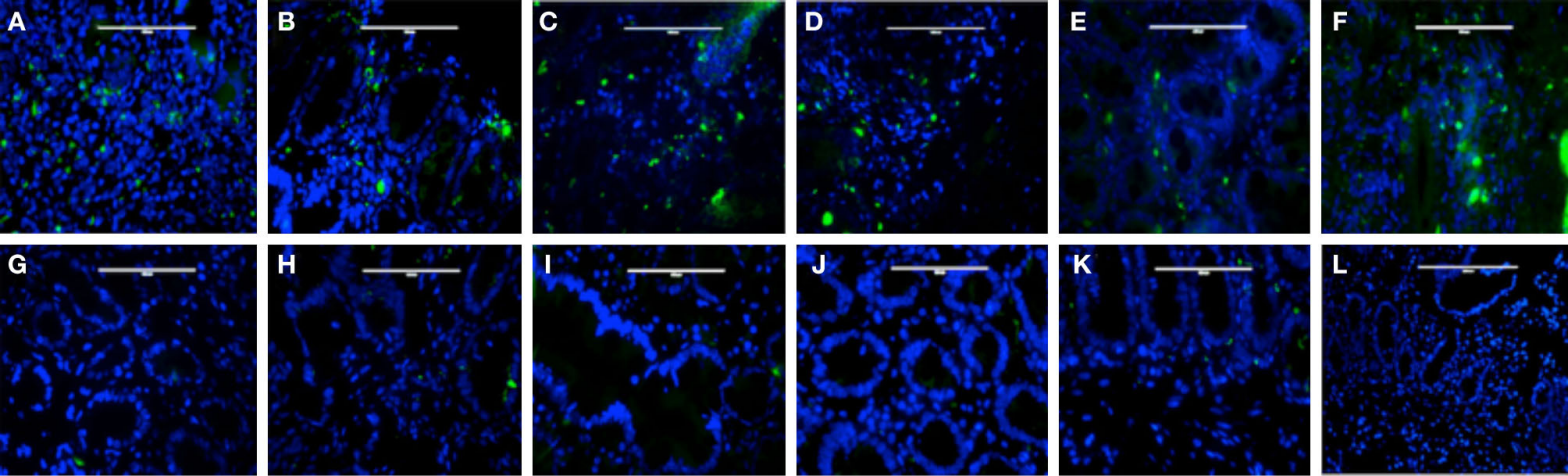
**Infiltration of plasmacytoid dendritic cells in duodenum biopsies of six subjects with HIV/AIDS (A–F) and six non-HIV subjects who were evaluated for gastritis (G–L)**. Biopsies were probed with antibodies FITC-anti-CD303 monoclonal antibody (green) and nucleus localization was determined by TOPO3 staining (blue). Bar represents 20 μm.

**Figure 3:**
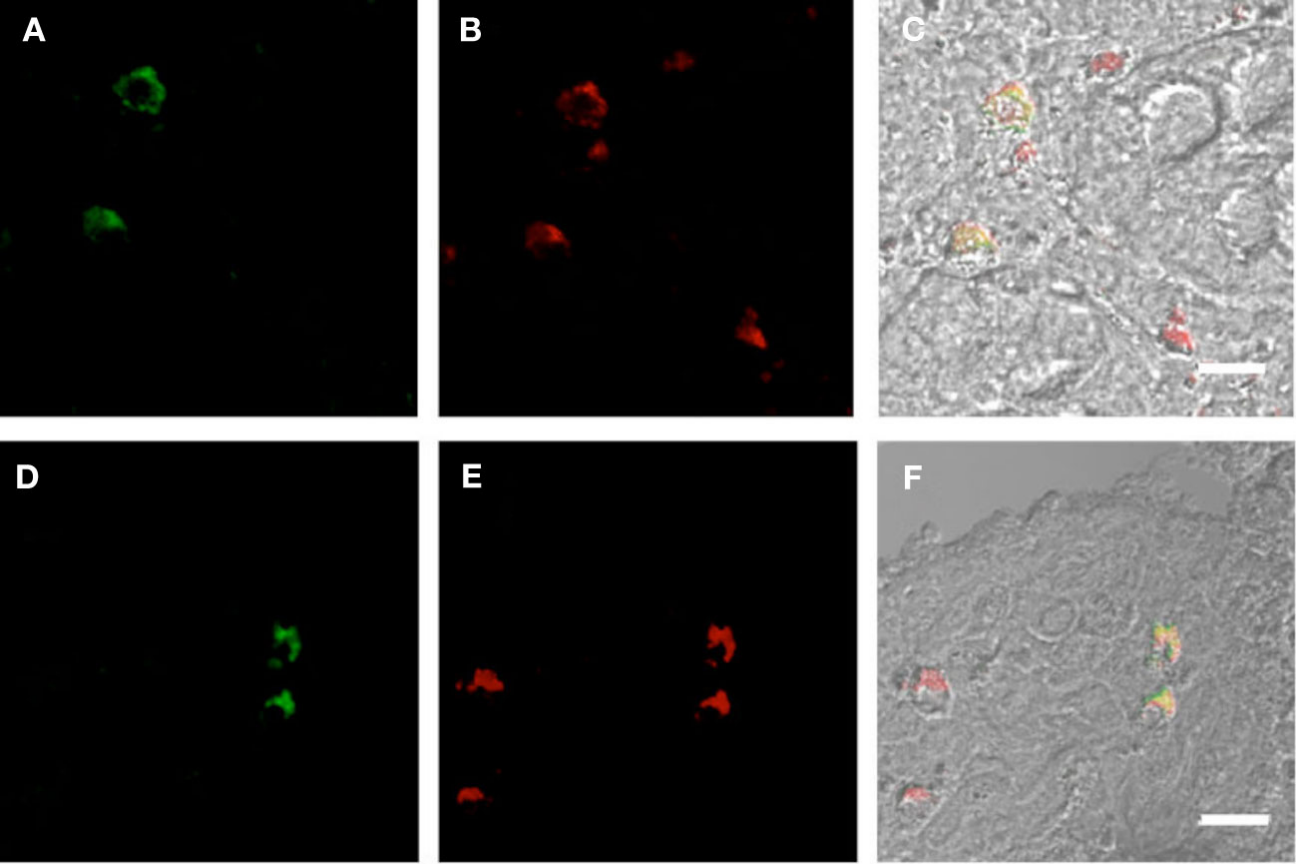
**Duodenum biopsies of HIV/AIDS cases and controls evaluated for coexpression of CD303 (green) and CD4 (red)**. Bar represents 20 μm. **(A)** HIV case duodenum biopsies probed with a monoclonal FITC-anti-CD303 antibody. **(B)** HIV case duodenum biopsies probed with a monoclonal PE-anti-CD4 antibody. **(C)** HIV case duodenum biopsies DIC image merged with **(A,B)**. **(D)** Control duodenum biopsies probed with a monoclonal FITC-anti-CD303 antibody. **(E)** Control duodenum biopsies probed with a monoclonal PE-anti-CD4 antibody. **(F)** Control duodenum biopsies DIC image merged with **(D,E)**.

**Figure 4:**
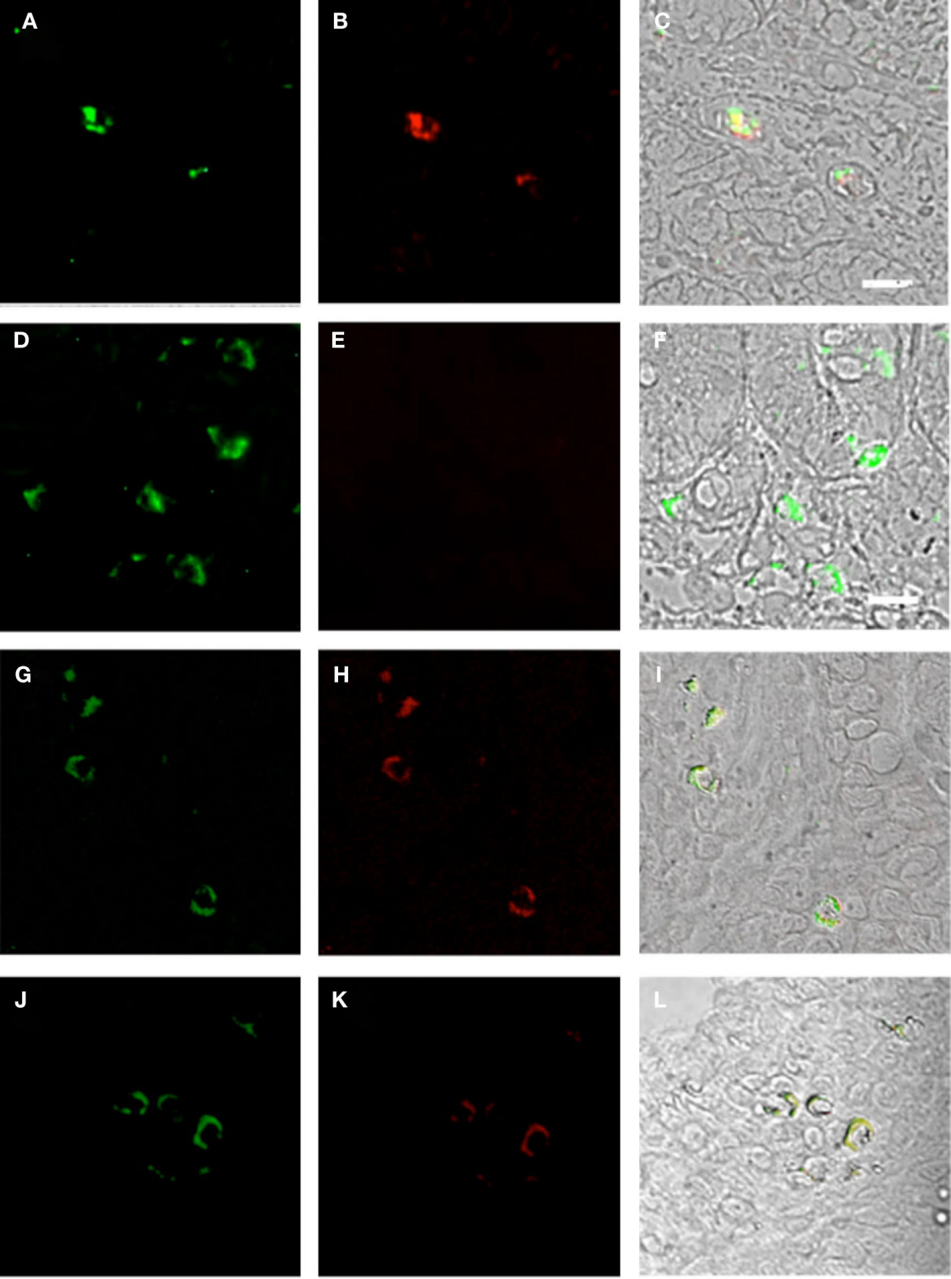
**Duodenum biopsies of HIV/AIDS cases and controls evaluated for coexpression of CD303 (green) and CD123 (red) and for CD303 (green) and CD80 (red)**. Bar represents 20 μm. **(A)** Control duodenum biopsies probed with a monoclonal FITC-anti-CD303 antibody. **(B)** Control duodenum biopsies probed with a monoclonal PE-anti-CD123 antibody. **(C)** Control duodenum biopsies DIC image merged with **(A,B)**. **(D)** HIV case duodenum biopsies probed with a monoclonal FITC-anti-CD303 antibody. **(E)** HIV case duodenum biopsies probed with a monoclonal PE-anti-CD123 antibody. **(F)** HIV case duodenum biopsies DIC image merged with **(D,E)**. **(G)** Control duodenum biopsies probed with a monoclonal FITC-anti-CD303 antibody. **(H)** Control duodenum biopsies probed with a monoclonal PE-anti-CD80 antibody. **(I)** Control duodenum biopsies DIC image merged with **(G,H)**. **(J)** HIV case duodenum biopsies probed with a monoclonal FITC-anti-CD303 antibody. **(K)** HIV case duodenum biopsies probed with a monoclonal PE-anti-CD80 antibody. **(L)** HIV case duodenum biopsies DIC image merged with **(J,K)**.

Plasmacytoid dendritic cells exclusively express the surface marker CD303 (BDCA-2) and non-exclusively express CD123, the receptor for the myeloid stimulatory cytokine IL-3 (26, 28, 29). Consistent with ability to become infected by HIV, they also express the HIV receptor CD4 as well as the coreceptors CCR5 and CXCR4. As with other antigen-presenting cells, when activated, they additionally express the B7 costimulatory molecules CD80 and CD86. Therefore, in order to characterize gut-associated pDCs in HIV/AIDS cases and controls, we probed the respective duodenum biopsies with fluorescently labeled anti-CD303, anti-CD123, anti-CD4, and anti-CD80 monoclonal antibodies (30). Consistent with previous reports, we observed a substantial infiltration of CD303^+^ pDCs in all duodenum biopsies from the HIV-infected subjects; however, we observed significantly fewer infiltrating pDCs in the control biopsies (Figure 2). In order to quantify these differences, five microscopic fields were randomly chosen for each subjects in each cohort and used to calculated average pDC infiltration (Table 2). We observed a sixfold greater infiltration of pDCs in the gut biopsies of HIV-infected cases over that of the controls (*p* < 0.0051). In order to further characterize the pDC infiltrate, double staining was conducted which confirmed that all CD303^+^ cells were also all CD4^+^ consistent with peripheral and pDCs and state II and III BM-derived pDCs (Figure 3). Additionally, all CD303^+^ cells were also CD123^+^ in the control biopsies (Figures 4A–C); however and unexpectedly, the CD123 staining was observably lower or absent for the HIV cohort (Figures 4D–F). Finally, double staining for CD303 and CD80 revealed that most of the CD303^+^ cells in the control biopsies also expressed the activation marker CD80 (Figures 4G–I), and all CD303^+^ cells in biopsies from the HIV cohort were also CD80^+^ (Figures 4J–L).

**Table 2:** **Quantitative analysis of plasmacytoid dendritic cells (pDCs)**.

Field	Controls						Mean/SD
	1	2	3	4	5	6	
1	1	1	2	3	1	2	
2	3	2	4	3	0	4	
3	0	5	1	1	3	2	
4	1	1	0	3	2	5	
5	1	1	2	1	2	2	
Sum	6	10	9	11	8	15	9.8/3.1

**Field**	**HIV cases**						**Mean/SD**
	**1**	**2**	**3**	**4**	**5**	**6**	

1	10	12	15	9	9	18	
2	12	7	14	15	14	18	
3	16	12	17	10	17	10	
4	10	15	10	13	11	13	
5	11	11	7	17	13	11	
Sum	59	57	63	64	64	70	61.4/4.5

*Five microscopic fields were chosen at random and used to count total pDCs in the duodenum of six controls (top) and six cases (bottom). *p* = 0.0051 by Mann–Whitney*.

### Gut-associated pDCs in HIV cases display an immature phenotype

**Figure 5:**
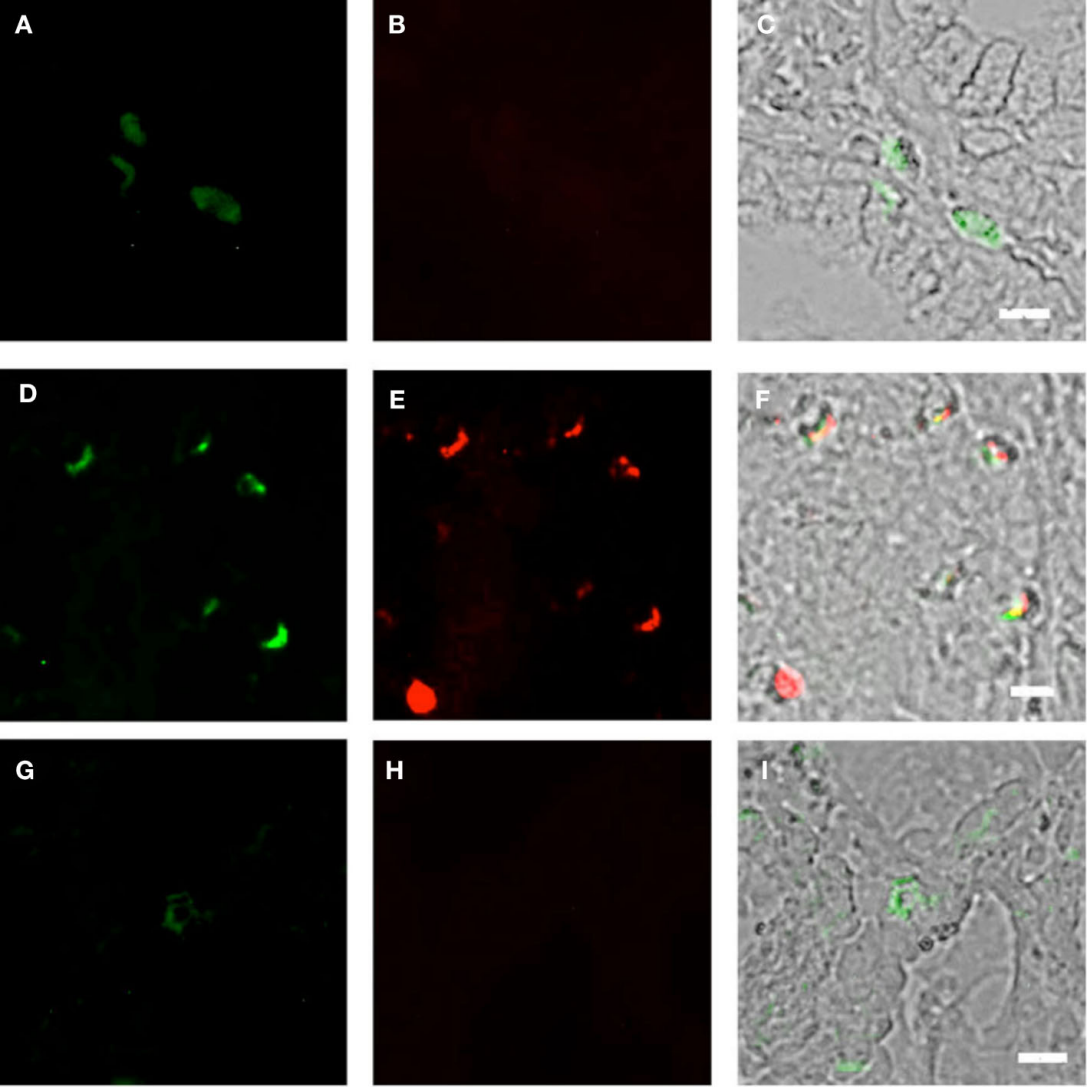
**Duodenum biopsies of HIV/AIDS cases and controls evaluated for coexpression of CD303 (green) and Ki-67 (red)**. Bar represents 20 μm. **(A)** Control duodenum biopsies probed with a monoclonal FITC-anti-CD303 antibody. **(B)** Control duodenum biopsies probed with a monoclonal PE-anti-Ki-67 antibody. **(C)** Control duodenum biopsies DIC image merged with **(D,E)**. **(D)** HIV case duodenum biopsies probed with a monoclonal FITC-anti-CD303 antibody. **(E)** HIV case duodenum biopsies probed with a monoclonal PE-anti-Ki-67 antibody. **(F)** HIV case duodenum biopsies DIC image merged with **(A,B)**. **(G)** HIV case duodenum biopsies probed with a monoclonal FITC-anti-CD303 antibody. **(H)** HIV case duodenum biopsies probed with a PE-Mouse IgG1,κ isotype control antibody. **(I)** HIV case duodenum biopsies DIC image merged with **(G,H)**.

**Figure 6:**
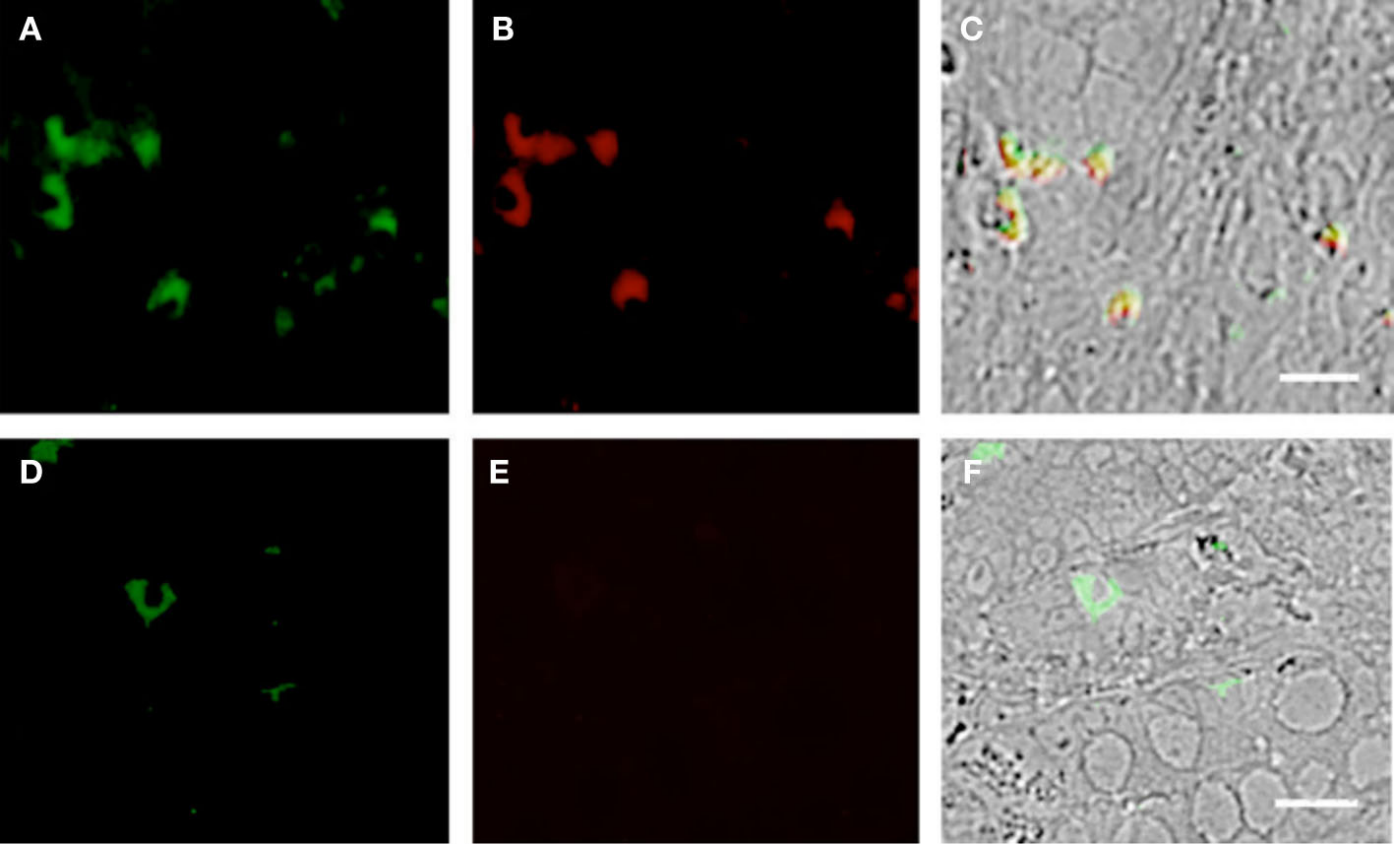
**Duodenum biopsies of HIV/AIDS cases and controls evaluated for coexpression of CD303 (green) and CD11c (red)**. Bar represents 20 μm. **(A)** HIV case duodenum biopsies probed with a monoclonal FITC-anti-CD303 antibody. **(B)** HIV case duodenum biopsies probed with monoclonal APC-anti-CD11c antibody. **(C)** HIV case duodenum biopsies DIC image merged with **(A,B)**. **(D)** Control duodenum biopsies probed with monoclonal FITC-anti-CD303 antibody. **(E)** Control duodenum biopsies probed with monoclonal APC-anti-CD11c antibody. **(F)** Control duodenum biopsies DIC image merged with **(D,E)**.

Using SIV-infected cynomolgus macaques, an animal model of HIV, Bruel and coworkers observed that gut-associated pDCs express the cellular proliferation marker Ki-67 (24). In that pDCs in the periphery do not divide, this observation suggests that the gut-associated pDCs in the SIV-infected macaques are pDCs precursors from bone marrow and not mature pDCs from the periphery. To the best of our knowledge, this observation has not been confirmed in humans. Therefore, we used fluorescently labeled anti-Ki-67 monoclonal antibodies to probe the biopsies of HIV-infected cases and controls. Consistent with the observations of Bruel et al., we observed CD303^+^/Ki-67^+^ cells in the biopsies of HIV-infected subjects (Figures 5A–C) but not in the control biopsies (Figures 5D–F). In order to evaluate the specificity of the primary antibody, we probed the same biopsies with a matched isotype control. An absence of non-specific staining (Figures 5G–I) confirmed that the immunoreactivity for CD303 and Ki-67 was indeed specific. A minor population of BM-derived pre-pDCs are also known to express the myeloid DC marker CD11c. Again, consistent with BM-derived pre-pDCs, we observed that most of the duodenum-associated pDCs in the HIV biopsies were also CD11c^+^ (Figures 6A–C); however, no CD303^+^/CD11c^+^ cells were observed in the control biopsies (Figures 6D–F).

### Gut-associated pDCs in HIV-infected subjects display a killer-pDC phenotype

**Figure 7:**
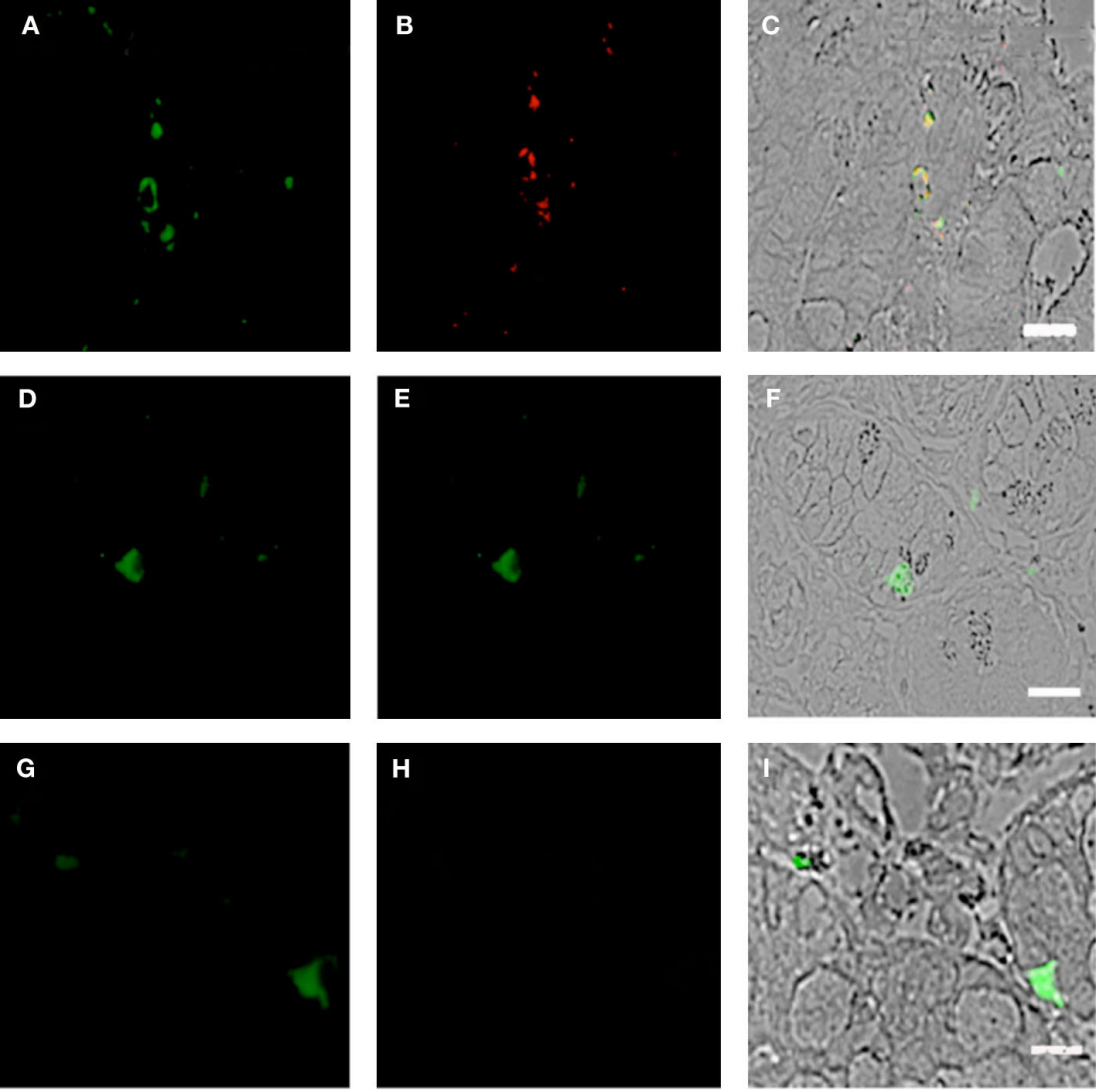
**Duodenum biopsies of HIV/AIDS cases and controls evaluated for coexpression of CD303 (green) and granzyme B (GZMB) (red)**. Bar represents 20 μm. **(A)** HIV case duodenum biopsies probed with a monoclonal FITC-anti-CD303 antibody. **(B)** HIV case duodenum biopsies probed with monoclonal APC-anti-GZMB antibody. **(C)** HIV case duodenum biopsies DIC image merged with **(A,B)**. **(D)** Control duodenum biopsies probed with monoclonal FITC-anti-CD303 antibody. **(E)** Control duodenum biopsies probed with monoclonal APC-anti-GZMB antibody. **(F)** Control duodenum biopsies DIC image merged with **(D,E)**. **(G)** HIV case duodenum biopsies probed with a monoclonal FITC-anti-CD303 antibody. **(H)** HIV case duodenum biopsies probed with a PE-Mouse IgG1 isotype control antibody. **(I)** HIV case duodenum biopsies DIC image merged with **(G,H)**.

**Figure 8:**
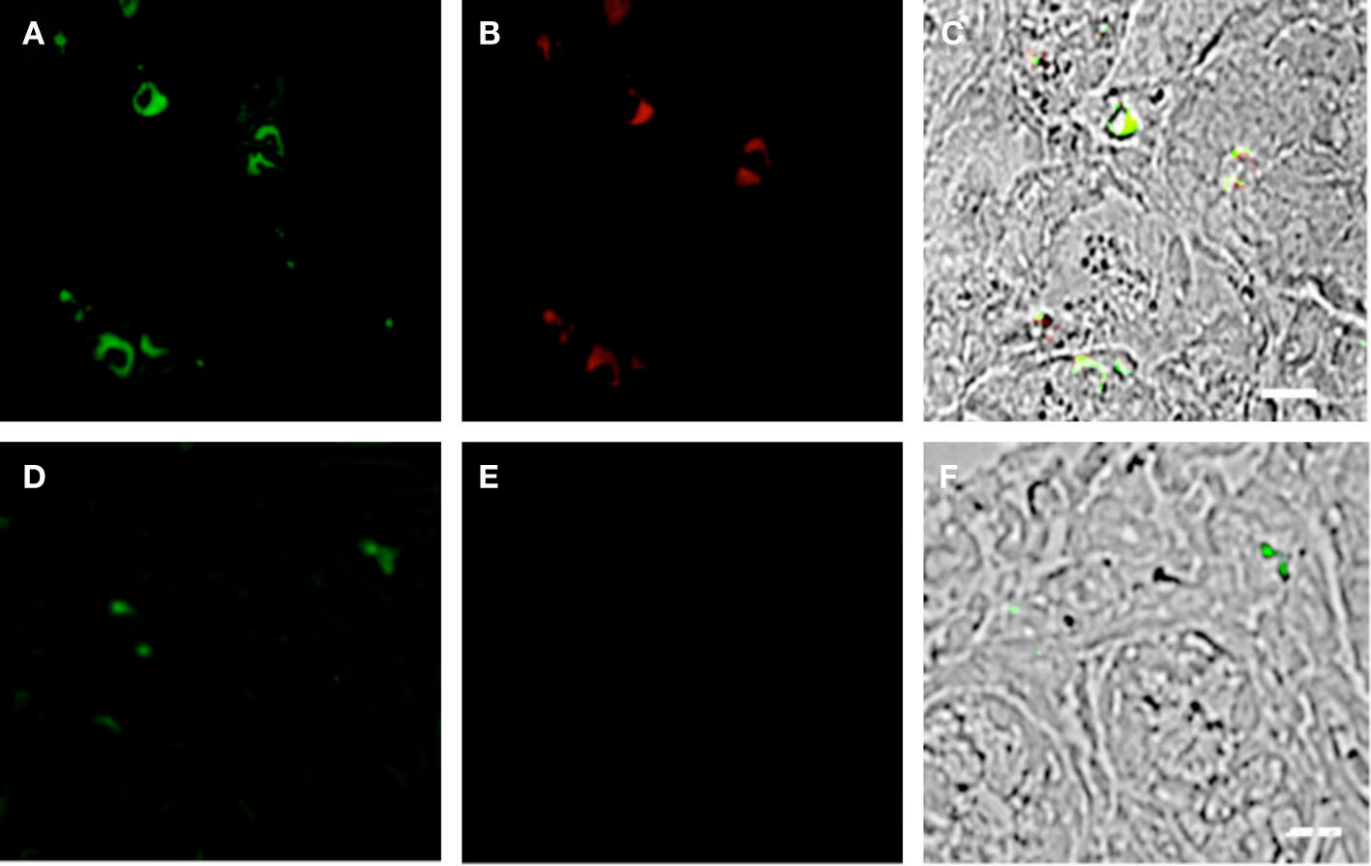
**Duodenum biopsies of HIV/AIDS cases and controls evaluated for coexpression of CD303 (green) and CD56 (red)**. Bar represents 20 μm. **(A)** HIV case duodenum biopsies probed with a monoclonal FITC-anti-CD303 antibody. **(B)** HIV case duodenum biopsies probed with monoclonal APC-anti-CD56 antibody. **(C)** HIV case duodenum biopsies DIC image merged with **(A,B)**. **(D)** Control duodenum biopsies probed with monoclonal FITC-anti-CD303 antibody. **(E)** Control duodenum biopsies probed with monoclonal APC-anti-CD56 antibody. **(F)** Control duodenum biopsies DIC image merged with **(D,E)**.

Previous studies have reported that the GI tract experiences significant pathology during the course of HIV infection [reviewed by Brenchley and Douek (31)]; however, the mechanism of this pathology is not fully understood. pDCs have the capacity to display a “killer” phenotype, with the ability to lyse target cells in either a GZMB- or TRAIL-dependent manner (32, 33). We therefore speculated that this may contribute to the gut mucosal damage associated with HIV infection. To this end, we probed the duodenum biopsies of HIV subjects and controls for coexpression of CD303 and GZMB and observed significant immunoreactivity (CD303^+^/GZMB^+^) in the HIV cohort (Figures 7A–C). In contrast, an absence of immunoreactivity with the anti-GZMB antibody was observed in control biopsies (Figures 7D–F). It has also been reported that GZMB-dependent killer pDCs also upregulate the neural adhesion marker CD56; therefore, we additionally probed the same biopsies with anti-CD56 and observed a number of CD303^+^/CDCD56^+^ cells in the biopsies of HIV/AIDS cases (Figures 8A–C). In contrast, no CD303^+^/CD56^+^ cells were observed in the controls biopsies (Figures 8D–F). Absence of reactivity with matched isotype controls for the anti-GZMB and CD56 antibodies suggested that the binding was specific.

## Discussion

As the principal source of type I IFN produced by circulating immune cells, pDCs play a seminal role in the innate antiviral immune response. Under homeostatic conditions, pDCs are primarily found in circulation and mucosal tissues such as the gastrointestinal lymphoid tissue (GALT) and the respiratory tract (34–36). Only small numbers of pDCs are normally present in peripheral tissue; however, their transmigration increases significantly during inflammation (37, 38). Once activated, pDCs have the capacity to release proinflammatory cytokines such as CXCL8 and CXCL10, and therefore, they also have the capacity to contribute to pathology.

Numerous studies have shown that pDCs are qualitatively and quantitatively impacted during HIV infection. For example, Malleret et al. reported a decline in the number of circulating pDCs in animals infected with SIV (17). Additionally, Brown and coworkers observed a rapid decline in circulating pDCs that correlated with their migration to the lymph nodes (19). Other studies have reported a decline in circulating pDC numbers in HIV cases that inversely correlated with viremia (16, 39, 40).

Several hypotheses have been proposed to explain the decrease in circulating pDCs associated with HIV infection, including, virus-associated cytopathic effects and pDC redistribution, from the circulation into tissue (9, 16, 41–44). Kwa and coworkers reported that pDCs infiltrate the colorectal region of the gut in SIV acutely infected rhesus macaques and that the gut infiltrating pDCs were producing high levels of IFNα and other proinflammatory cytokines (45). Additionally, Reeves et al. reported that SIV infection induces the accumulation of pDCs in the gut mucosa (23), and Li et al. observed a fourfold increase in pDC accumulation in jejunum, colon, and gut-draining LNs of SIV-infected rhesus macaques (46). Similar distributions of pDCs have been observed in HIV cases. For instance, Lehmann and coworkers observed increased pDC accumulation in the terminal ileum of HIV-infected subjects (47), and tissue homing was explained by significant upregulation of the gut-homing receptor CD103, when compared with uninfected controls. These data support a model whereby pDCs redistribute from the circulation into the gut during the course of HIV infection. Although the previous data unequivocally supported this supposition, it was recently challenged by the report of Bruel and colleagues whereby they described the depletion of IFN-producing pDCs in the periphery as a result of activation-driven exhaustion, followed by a concomitant increase in gut-associated pDCs (24). They further reported that the gut-associated pDCs are primarily naive Ki-67^+^ pDCs, suggesting that the BM-derived pDCs egressed from the BM to replace the depleted circulating pDCs and subsequently migrate from the periphery to the gut. Therefore, the current data suggest that the loss of peripheral pDCs during the course of HIV infection is not just a matter of tissue redistribution but a combination of pDC depletion, repopulation, and migration.

During the acute infection stage, pDCs respond with a robust IFN production; however as the acute stage transitions to the chronic stage, they become refractory with respect to their ability to produce IFN (48). BM-derived pDC precursors have little capacity to produce type I IFN and may also have a phenotype different from that of mature circulating pDCs that depends on their stage of development (27). Three subsets of pre-pDCs have been described based on the expression level of CD34 and HLA-DR (Class II) surface markers. All three populations express CD123 (IL-3α chain receptor); however, CD4 expression is absent in the earliest pre-pDCs (Stage I pre-pDCs) but becomes evident in the more developed pre-pDCs (Stages II and III) (27). Additionally, CD184 (CXCR4) is observed in all three stages. These observations suggest that pre-pDCs have the capacity to be infected by HIV at least as early as Stage II.

In the present study, we observed that duodenum-associated CD303^+^ pDCs expressed Ki-67, but no anti-Ki-67 immunoreactivity was observed in the control biopsies. These observations are consistent with those made in SIV-infected cynomologus macaques by Bruel et al. (24). Ki-67 is nuclear proliferation antigen, which is expressed by cells in a non-G_0_ phase of the cell cycle and therefore is indicative of pDCs that were recently mobilized from the bone marrow (19, 49). Additionally, CD303 is not present on Stage I BM-derived pre-pDCs (27); therefore, our data further suggests that duodenum-associated pDCs in HIV-infected subjects are largely consistent with Stage II or Stage III pre-pDCs. We also observed that gut-associated CD303^+^ pDCs expressed the B7 costimulatory molecule CD80. The B7 costimulatory molecule CD80 is upregulated during *in vitro* HIV infection of pDCs (50); however, circulating pDCs from healthy or HIV-infected subjects show little, if any, CD80 expression. Additionally, characterization of pre-pDCs from healthy BM-donors suggests that these cells do not express CD80 (27). Our observation of CD80 expression by gut-associated pDCs implies that these cells may be activated preferentially in the gut over that of those in the circulation. Also, in contrast to controls, we observed that gut-associated pDCs in HIV-infected subjects express the common myeloid dendritic cell protein CD11c. Although murine pDCs express CD11c at low levels, its expression is typically absent on circulating human pDCs. Notwithstanding, CD11c expressed on a subpopulation of BM-derived pre-pDCs has been described, again supporting the supposition that the gut-associated pDCs in our HIV cohort are naive BM-derived pDCs.

Activated pDCs have the capacity to express the cytotoxic enzyme GZMB; however, its expression is downregulated in the presence of IFNα and upregulated by IL-3 (51). Not only are BM-derived pre-pDCs refractory to the production of type I IFN, we observed gut-associated pDCs in our HIV cohort have low or absent CD123 expression, albeit this observation is only qualitative in that it was not practical to quantify this value by IHC. IL-3 signaling and GZMB expression is intimately connected (51); therefore, a dysregulation in CD123 (the IL-3 receptor) may potentially impact the expression of GZMB. In addition to the cytotoxic and proapoptotic role of GZMB, it also plays a role in inflammation and tissue remodeling (50). Severe tissue damage leading to increased intestinal epithelium permeability is hallmark of HIV infection (52), and this process is not reversed even after long-term HAART therapy (53–55). Although speculative at this point, the presence of GZMB may suggest a potential mechanism that contributes to the decreased regenerative capacity of the gut epithelium and increased mucosal permeability and inflammation associated with HIV infection (56–58). To the best of our knowledge, the expression of GZMB and the downregulation of CD123 in gut-associated pDCs have not been described in HIV; however, future studies will be required to fully elucidate the role of GZMB in HIV-associated gut pathology. Finally, we observed that most CD303^+^ cells also expressed the neural adhesion molecule CD56. Tel et al. have previously described the coexpression of CD303, CD56, and GZMB by pDCs activated with the preventative vaccine to tick-borne encephalitis virus FSME. These “killer pDCs” possessed the tumoricidal capacity to lyse K562 and Daudi cells in a contact-dependent manner (59). They additionally reported that CD303 and CD56 expression coincided with elevated expression of programmed death-ligand 1 (PD-L1), GZMB, and TNF-related apoptosis-inducing ligand (TRAIL). It is also noteworthy that all pDC neoplasms express the CD56 marker (60), further suggesting that CD56 expression by pDCs is not necessarily novel or without precedence. The significance of this marker has yet to be determined on NK cells so the contribution to pDC biology in the context of HIV infection will require further investigations as well.

## Conclusion

In summary, our data show that subjects with HIV have, on average, decreased pDCs in the periphery, when compared with healthy controls, consistent with previous reports. Additionally, we show that gut-associated pDCs in HIV cases express the cellular proliferation marker Ki-67, which suggests that the gut-associated pDCs are naive and likely of bone marrow origin. Finally, we observed that gut-associated pDCs have an activated phenotype and also upregulate the proapoptotic enzyme GZMB. When taken together, our data support a model of HIV progression whereby circulating pDCs are depleted and replaced by naive BM-derived pDCs, which have little, if any, IFN-producing capacity. Ultimately, these pDCs migrate to the gut, potentially subjecting the gut mucosa to the inflammatory effects and damage associated with inflammatory cytokine production and GZMB expression. The type I IFN produced by competent pDCs is a critical part of an innate immune response to viral infection. Therefore, a greater understanding of the fate of these cells in HIV infection may lead to strategies that can restore the IFN-producing capacity of the innate immune system.

## Conflict of Interest Statement

The authors declare that the research was conducted in the absence of any commercial or financial relationships that could be construed as a potential conflict of interest.

## Supplementary Material

The Supplementary Material for this article can be found online at http://journal.frontiersin.org/article/10.3389/fimmu.2015.00485
