# Supplementary material for: Gut-Associated Plasmacytoid Dendritic Cells Display an Immature Phenotype and Upregulated Granzyme B in Subjects with HIV/AIDS
Source: Front Immunol. 2015 Sep 24;6:485. doi: 10.3389/fimmu.2015.00485 (PMC4585323; doi:10.3389/fimmu.2015.00485)
Supplement: Supplementary file 1 [file Table_1.DOCX]

**Supplemental Table 1.** Peripheral pDC levels with respect to CD4 count and viral load in treated and untreated HIV^+^ subjects.

| **Peripheral pDCs**  **(CD303/CD45)** | **Average**  **CD4 count**  **(cells/mm^3^)** | **Average viral**  **Load**  **Copies/mL** | **Antiviral**  **treatment** | **Months since initial diagnosis** |
| --- | --- | --- | --- | --- |
| <0.01 | 3 | 257800 | 3 months | 1 year |
| 0.01-0.05 | 22 | 113500 | no | 2 months |
| 0.01-0.05 | 30 | 34700 | yes | unkonwn |
| 0.01-0.05 | 59 | 56000 | 4 months | 3 years |
| 0.01-0.05 | 88 | 639000 | no | 3 months |
| 0.01-0.05 | 33 | 590300 | 5 months | 8 years |
| 0.01-0.05 | 6 | 420800 | 6 months | 9 years |
| 0.01-0.05 | 34 | 784700 | 2 months | 12 years |
| 0.05-0.10 | 72 | 0 | 9 months | 10 years |
| 0.05-0.10 | 867 | 0 | 5 month | 12 years |
| 0.05-0.10 | 788 | 142100 | no | 2 months |
| 0.05-0.10 | 55 | 762500 | no | 3 years |
| 0.05-0.10 | 91 | 2400 | 15 months | 10 years |
| 0.05-0.10 | 410 | 0 | 10 months | 1 year |
| 0.05-0.10 | 54 | 2600 | 36 months | 12 years |
| 0.05-0.10 | 346 | 233900 | no | 2 years |
| 0.05-0.10 | 384 | 0 | 6 months | 6 years |
| 0.05-0.10 | 205 | 43 | no | 3 month |
| >0.10 | 509 | 0 | 5 months | 10 years |
| >0.10 | 285 | 330 | 6 months | 3 years |
| >0.10 | 901 | 1500 | no | 1 year |
| >0.10 | 371 | 45400 | 2 month | 13 years |
| >0.10 | 455 | 0 | 6 months | 7 years |
